# ADLD: A Novel Graphical Representation of Protein Sequences and Its Application

**DOI:** 10.1155/2014/959753

**Published:** 2014-10-30

**Authors:** Lei Wang, Hui Peng, Jinhua Zheng

**Affiliations:** ^1^Key Laboratory of Intelligent Computing & Information Processing, Ministry of Education, Xiangtan University, Xiangtan 411105, China; ^2^College of Information Engineering, Xiangtan University, Xiangtan 411105, China

## Abstract

To facilitate the intuitional analysis of protein sequences, a novel graphical representation of protein sequences called ADLD (*Alignment Diagonal Line Diagram*) is introduced in this paper first, and then a new ADLD based method is proposed and utilized to analyze the similarity/dissimilarity of protein sequences. Comparing with existing methods, our ADLD based method is proved to be effective in the similarity/dissimilarity analysis of protein sequences and have the merits of good intuition, visuality, and simplicity. The examinations of the similarities/dissimilarities for both the 16 different ND5 proteins and the 29 different spike proteins illustrate the utility of our ADLD based approach.

## 1. Introduction

Homology analysis is one of the hot topics in the area of protein sequences analysis. Up to now, lots of methods have been proposed for the homology analysis of protein sequences [1–3], and among them a useful one is the graphical representation of protein sequences, which is proved to be a powerful tool for visual comparison of protein sequences.

At first, graphical representation methods were introduced for representation of DNA sequences on the basis of multiple dimension space [4–7]. After obtaining the sequence invariants from the graphics, one can compare the sequences based on comparison of sequence invariants. Graphical representation methods were proposed as an alternative approach of direct comparison of DNA sequences, which are computational intensive (even those of a restricted length) [8]. Protein sequences are to some degree similar to DNA sequences, which are composed of different units. Thus the graphical representation methods can be extended to describe protein sequences obviously.

Currently, many researchers have proposed different methods for the graphical representation of protein sequences [9–24]. For example, Feng and Zhang [25] suggested Zp-curve based on the hydrophobicity and charged properties of amino acid residues along the primary sequence. Randić et al. [26] introduced a graphical representation of protein sequences based on a graphical representation of triplets of DNA in which the interior of a square or a tetrahedron is utilized to accommodate 64 sites for the 64 codons. Bai and Wang [27] derived a 2D graphical representation of protein sequences based on nucleotide triplet codons. Yao et al. [28] outlined a 2D graphical representation of protein sequences based on two classifications of amino acids. Abo el Maaty et al. [29] proposed a novel unique 3D graphical representation of protein sequences based on three physicochemical properties of amino acid side chains. Abo-Elkhier introduced a 3D graphical representation of protein sequence based on a right cone of a unit base and unit height on protein sequences interfaces [30]. El-Lakkani and El-Sherif [31] proposed a graphical representation of protein sequence to help similarity analysis of protein sequences based on 2D and 3D amino acid adjacency matrices. Ma et al. [32] introduced a family of Iterated Function Systems (IFS) to outline a 2D graphical representation of protein sequences.

In most of these existing methods, the main drawbacks are that the higher the dimension of the protein sequence graphs, the heavier the computation complexity of the methods or the lower the recognition degree of the protein sequence graphs. For example, in the methods proposed in [26, 28], the main drawback is that the lines will cross each other, which will decrease the visibility of the graphics. In the methods proposed in [29–31], the main drawbacks are that the 3D graphics seem to be more complex and have lower visibility than the 2D graphics, and, in addition, to obtain the sequence invariants from the graphics, complex matrixes are required to be constructed, which need much computation and storage.

Sequence alignment is a way of arranging the sequences of DNA, RNA, or protein to identify regions of similarity that may be a consequence of functional, structural, or evolutionary relationships between the sequences [33]. Up to now, there are many kinds of algorithms having been implemented for sequence alignment [34–37]. These methods are usually efficient but complex and time consuming. Comparing with the alignment methods, existing graphical representation methods can also display the inner structure of the protein sequences and can be utilized to find the similarity/dissimilarity more visible according to their graphics. In this paper, we proposed a novel method for analyzing the similarity/dissimilarity by combining the idea of the sequence alignment and the graphical representation methods to some degree avoid the weakness of both of these two methods.

Principal components analysis (PCA) is a standard tool in multivariate data analysis to reduce the number of dimensions, which has been proved to be effective in the process of protein sequence analysis [38–40]. Therefore, in order to overcome the main drawbacks of existing methods, in this paper, a novel graphical representation of protein sequences called ADLD (*Alignment Diagonal Line Diagram*) is introduced based on PCA, and then a new ADLD based method is proposed and utilized to analyze the similarity/dissimilarity of protein sequences. And, in addition, to validate the effectiveness of our ADLD based method, we adopt it to analyze the similarity/dissimilarity of both the 16 different ND5 proteins and the 29 different spike proteins, respectively, which are widely used as the test data [16–26]. The analysis results show that our method is not only visual, intuitional, and effective in the similarity/dissimilarity analysis of protein sequences but also quite simple, since there are no high dimensional matrixes required to be constructed.

## 2. Materials and Methods

### 2.1. Procedure of Our Method for Analysis of Protein Sequences

In this section, we will illustrate the overall procedures of our method for analyzing protein sequences as follows at first.

1. Select the same 9 different properties for each amino acid and construct a 20 × 9 matrix as the input data of the PCA algorithm on the basis of total 20 different amino acids.
2. According to the PCA algorithm, we can obtain a unique feature for each amino acid.
3. For each protein sequence in the test data, we will replace each amino acid in the protein sequence with its corresponding unique feature, and then we can transform the protein sequence into a numerical sequence.
4. For any two numerical sequences, we can draw a graph, named ADLD, and then abstract some numerical characteristics of it, which can be utilized to analyze the similarity/dissimilarity of these two sequences.

Next, in Sections 2.2–2.6 we will introduce the details of constructing the ADLDs and obtaining some of the numerical characteristics of them. In Section 3.1, we will give the method for constructing the similarity/dissimilarity of our test sequence groups.

### 2.2. Amino Acids and Their Properties

Proteins are composed of 20 different amino acids, and these amino acids have many different physicochemical and biological properties such as the molecular weight (mW), hydropathy index (hI), the pKa value for terminal amino acid groups COOH (pK1), the pKa value for terminal amino acid groups NH_3_^+^ (pK2), isoelectric point (pI), solubility (*S*), the number of triplet codons (cN), frequency of human proteins (*F*), and van der Waals radius of side chains (vR). The names and symbols of the 20 amino acids and the value of their 9 major properties are illustrated in Table 1.

### 2.3. Principal Components Analysis

*Principal components analysis* (PCA) is a common technique for dimensionality reduction and pattern recognition in datasets of high dimension [41]. The main purposes of PCA are the analysis of data to identify patterns and finding patterns to reduce the dimensions of the dataset with minimal loss of information. The general steps of conducting PCA are as follows.

**Step 1 .**

For *m* samples {*X*_1_, *X*_2_,…, *X*_*m*_}, suppose that each *X*_*i*_ has *n* components {*x*_*i*1_, *x*_*i*1_,…, *x*_*in*_}, let *X*_*i*_ = (*x*_*i*1_, *x*_*i*1_,…, *x*_*in*_) for *i* ∈ {1,2,…, *m*}, and then construct an *m* × *n* matrix **X** according to the following formula first:

(1) $$\begin{array}{c} X=\left[\begin{array}{c} X_{1}\\ X_{2}\\ \vdots \\ X_{m} \end{array}\right]=\left[\begin{array}{cccc} x_{11} & x_{12} & \cdots & x_{1n}\\ x_{21} & x_{22} & \cdots & x_{2n}\\ \vdots & \vdots & \vdots & \vdots \\ x_{m1} & x_{m2} & \cdots & x_{mn} \end{array}\right]. \end{array}$$

Next, based on the matrix **X**, construct the corresponding *m* × *n* standardized matrix **X** ^*^ according to the following formula:

(2) $$\begin{array}{c} X^{*}=\left[\begin{array}{c} X_{1}^{*}\\ X_{2}^{*}\\ \vdots \\ X_{m}^{*} \end{array}\right]=\left[\begin{array}{cccc} x_{11}^{*} & x_{12}^{*} & \cdots & x_{1n}^{*}\\ x_{21}^{*} & x_{22}^{*} & \cdots & x_{2n}^{*}\\ \vdots & \vdots & \vdots & \vdots \\ x_{m1}^{*} & x_{m2}^{*} & \cdots & x_{mn}^{*} \end{array}\right], \end{array}$$

where *X*_i_^*^ = (*x*_*i*1_^*^, *x*_*i*2_^*^,…, *x*_in_^*^), $x_{ij}^{*}=\left(x_{ij}-{\overset{-}{x}}_{j}\right)/\sqrt{\text{var}(x_{j})}$, ${\overset{-}{x}}_{j}=\left(1/n\right)\sum_{i=1}^{n}x_{ij}$, and $\text{var}(x_{j})=\left(1/\left(n-1\right)\right)\sum_{i=1}^{n}{\left(x_{ij}-{\overset{-}{x}}_{j}\right)}^{2}$, for *i* ∈ {1,2,…, *m*} and *j* ∈ {1,2,…, *n*}.

**Step 2 .**

Based on the matrix **X** ^*^, construct the *n* × *n* correlation matrix **R** according to the following formula:

(3) $$\begin{array}{c} R=\left[\begin{array}{c} R_{1}\\ R_{2}\\ \vdots \\ R_{n} \end{array}\right]=\left[\begin{array}{cccc} r_{11} & r_{12} & \cdots & r_{1n}\\ r_{21} & r_{22} & \cdots & r_{2n}\\ \vdots & \vdots & \vdots & \vdots \\ r_{n1} & r_{n2} & \cdots & r_{nn} \end{array}\right], \end{array}$$

where we can find that $r_{ij}=\sum_{k=1}^{m}\left(x_{ki}^{*}-{\overset{-}{x}}_{i}^{*})(x_{kj}^{*}-{\overset{-}{x}}_{j}^{*}\right)/\sqrt{\sum_{k=1}^{m}{\left(x_{ki}^{*}-{\overset{-}{x}}_{i}^{*}\right)}^{2}\sum_{k=1}^{m}{\left(x_{kj}^{*}-{\overset{-}{x}}_{j}^{*}\right)}^{2}}$ for *i* ∈ {1,2,…, *n*} and *j* ∈ {1,2,…, *n*}.

**Step 3 .**

From the correlation matrix **R**, obtain its *n* eigenvalues *λ*_1_ ≥ *λ*_2_ ≥ ⋯≥*λ*_*n*_ > 0 and the corresponding *n* eigenvectors

(4) $$\begin{array}{c} a_{1}=\left[\begin{array}{c} a_{11}\\ a_{21}\\ \vdots \\ a_{n1} \end{array}\right],a_{2}=\left[\begin{array}{c} a_{12}\\ a_{22}\\ \vdots \\ a_{n2} \end{array}\right],\ldots ,a_{n}=\left[\begin{array}{c} a_{1n}\\ a_{2n}\\ \vdots \\ a_{nn} \end{array}\right], \end{array}$$

respectively. And, from now on, we can obtain *n* principal components *F*_*i*_ for *i* ∈ {1,2,…, *n*} as follows:

(5) $$\begin{array}{c} F_{i}=a_{1i}X_{1}^{*}+a_{2i}X_{2}^{*}+\cdots +a_{ni}X_{n}^{*}. \end{array}$$

**Step 4 .**

For each principal component *F*_*i*_ for *i* ∈ {1,2,…, *n*}, obtain its* contribution rate *CR_*i*_ and* accumulated contribution rate *ACR_*i*_ according to the following formulas, respectively:

(6) $$\begin{array}{c} {\text{CR}}_{i}=\frac{\lambda_{i}}{\sum_{k=1}^{n}\lambda_{k}}, \end{array}$$

(7) $$\begin{array}{c} {\text{ACR}}_{i}=\overset{i}{\underset{k=1}{\sum }}{\text{CR}}_{k}. \end{array}$$

Generally, in order to lower the computation complexity, we can keep only the first *t* (*t* ≤ *n*) principal components {*F*_1_, *F*_2_,…, *F*_*t*_}, where the accumulated contribution rate of the *t*th principal component *F*_*t*_ shall satisfy the fact that ACR_*t*_ ≥ 85%.

**Step 5 .**

For *j* ∈ {1,2,…, *t*}, let

(8) $$\begin{array}{c} F_{j}=\left[\begin{array}{c} F_{1}^{j}\\ F_{2}^{j}\\ \vdots \\ F_{m}^{j} \end{array}\right]. \end{array}$$

Then, for each *i* ∈ {1,2,…, *m*}, we can obtain the total score of the *i*th sample as follows:

(9) $$\begin{array}{c} \text{TotalScore}\left(i\right)=\overset{t}{\underset{k=1}{\sum }}F_{i}^{k}\times {\text{CR}}_{k}. \end{array}$$

### 2.4. PCA of the Amino Acids

Observing Table 1, if we consider the 20 amino acids as 20 different samples and the 9 properties of each amino acid as its 9 components, then, according to the general steps of conducting PCA illustrated in Section 2.3, we can obtain a 20 × 9 matrix **X** and its standardized matrix **X** ^*^, a 9 × 9 correlation matrix **R**, and 9 principal components {*F*_1_, *F*_2_,…, *F*_9_}. And, therefore, as illustrated in Table 2, we can obtain the 9 eigenvalues of **R** and the contribution rates and the accumulative contribution rates of the 9 principal components {*F*_1_, *F*_2_,…, *F*_9_}, respectively.

From Table 2, we can see that the accumulative contribution rate of the first 4 principal components amounts to 0.8588 (=85.88%), which is already bigger than 85%. Therefore, we can keep the first 4 principal components only. Let {*λ*_1_, *λ*_2_, *λ*_3_, *λ*_4_} be the 4 eigenvalues corresponding to the first 4 principal components, respectively; then, as illustrated in Table 3, we can obtain the 4 eigenvectors {*a*_1_, *a*_2_, *a*_3_, *a*_4_} corresponding to the 4 eigenvalues {*λ*_1_, *λ*_2_, *λ*_3_, *λ*_4_} separately.

Based on Table 3, we can obtain the first 4 principal components {*F*_1_, *F*_2_, *F*_3_, *F*_4_} as follows:

(10) F1=0.5036X1∗−0.2454X2∗−0.1634X3∗−0.3101X4∗+0.0702X5∗−0.1665X6∗−0.3872X7∗−0.4377X8∗+0.4349X9∗,F2=0.1436X1∗−0.1875X2∗+0.1820X3∗−0.1883X4∗+0.6464X5∗+0.4465X6∗+0.3931X7∗+0.1844X8∗+0.2643X9∗,F3=0.0571X1∗+0.2304X2∗+0.6298X3∗−0.3964X4∗−0.0786X5∗−0.5280X6∗+0.1003X7∗+0.2544X8∗+0.1738X9∗,F4=0.2158X1∗+0.6547X2∗+0.2288X3∗+0.5071X4∗+0.0532X5∗+0.1877X6∗−0.0532X7∗−0.2273X8∗+0.3495X9∗.

Observing the above 4 formulas, it is easy to find that there are three big coefficients in the first formula, which are 0.5036 (corresponding to mW), 0.4377 (corresponding to *F*), and 0.4349 (corresponding to vR), respectively. Therefore, it means that the three properties such as mW, *F*, and vR will have a major role in the first principal component *F*_1_. Similarly, we can also know that the three properties such as pI, *S*, and cN will have a major role in the second principal component *F*_2_, the third principal component *F*_3_ is mainly determined by pK1 and *S*, and the fourth principal component *F*_4_ is closely linked with hI and pK2 and so forth. Hence, we can obtain the total scores of the 20 amino acids as illustrated in Table 4 according to formula (9).

### 2.5. Numerical Sequences of Protein Sequences

Let *Ω* = {A, C, D, E, F, G, H, I, K, L, M, N, P, Q, R, S, T, V, W, Y} and suppose that Ψ = *p*_1_ *p*_2_ *p*_3_ … *p*_*N*_ represents a protein sequence with *N* amino acids, where *p*_*i*_ ∈ *Ω* for *i* ∈ {1,2,…, *N*}; then we can obtain a numerical sequence *S*_Ψ_ = (*t*_1_, *t*_2_,…, *t*_*N*_) corresponding to the protein sequence Ψ through replacing each amino acid *p*_*i*_ in Ψ with its corresponding value of TotalScore(*i*) for *i* ∈ {1,2,…, *N*}.

For example, consider the following 3 abbreviated protein sequences:

 Hu = MTMHTTMTTL,
 Gor = MTMYATMTTL,
 Opo = MKVINISNTM.

According to the above descriptions and Table 4, then we can obtain their corresponding numerical sequences as follows:

(11) SHu=0.5735,−0.4525,0.5735,0.4476,−0.4525,−0.4525,0.5735,−0.4525,−0.4525,−0.1205;SGor=0.5735,−0.4525,0.5735,0.9050,−0.9324,−0.4525,0.5735,−0.4525,−0.4525,−0.1205;SOpo=0.5735,0.7868,−0.2643,0.1435,−0.0242,0.1435,−0.7077,−0.0242,−0.4525,0.5735.

### 2.6. ASDs and ADLDs of Protein Sequence Pairs

For a given protein sequence pair (*s*_1_, *s*_2_), suppose that the protein sequence *s*_1_ includes *N*_1_ amino acids, *s*_2_ includes *N*_2_ amino acids, and *N*_1_⩾*N*_2_; then, in order to measure the similarity/dissimilarity between them, in this section, we will present a new method called* Alignment Scatter Diagram* (ASD) to plot the two sequences into a scatter diagram first. And, for convenience, we call the points in the ASD the* alignment-plots* (APs). The ASD of the protein sequence pair (*s*_1_, *s*_2_) can be obtained through the following steps.

**Step 1 .**

According to the method given in Section 2.5, translate the protein sequence pair (*s*_1_, *s*_2_) into two numerical sequences with the same length as follows:

(12) $$\begin{array}{c} S_{1}=\left\{t_{1},t_{2},\ldots ,t_{N_{1}}\right\},\\ S_{2}=\left\{t_{1},t_{2},\ldots ,t_{N_{2}},\underset{N_{1}-N_{2}}{\underset{︸}{0,0,\ldots ,0}}\right\}. \end{array}$$

**Step 2 .**

Let *w* be the* alignment width* (AW) of the protein sequence pair (*s*_1_, *s*_2_); that is, let *s*_1_ = *p*_1_, *p*_2_, *p*_3_,…, *p*_*N*_1__, *s*_2_ = *q*_1_, *q*_2_, *q*_3_,…, *q*_*N*_2__; then, for any amino acid *p*_*i*_ in the protein sequence *s*_1_, we will compare it with these 2*w* + 1 amino acids {*q*_*i*−*w*_,…, *q*_*i*−1_, *q*_*i*_, *q*_*i*+1_,…, *q*_*i*+*w*_} in the protein sequence *s*_2_, and then *w* can be simply defined as follows:

(13) $$\begin{array}{c} w=\left\{\begin{array}{cc} \xi , & \text{if}\;\;N_{1}-N_{2}\leq \xi ,\\ N_{1}-N_{2}, & \text{else}, \end{array}\right. \end{array}$$

where *ξ* > 0 is a given threshold to guarantee that the AW of the protein sequence pair (*s*_1_, *s*_2_) will not be too small to expose the association of the inner structures of the protein sequence pair (*s*_1_, *s*_2_). In actual applications, we suggest that *ξ* shall be no less than 10.

**Step 3 .**

Let *ε* > 0 be the* dissimilarity degree* (DD) of two amino acids; that is, if *ε* = 0, then it means that the two amino acids are the same; otherwise, it means that the two amino acids are different from each other to some degree, and then the APs in the ASD of the protein sequence pair (*s*_1_, *s*_2_) can be briefly defined as follows:

(14) $$\begin{array}{c} A_{ij}^{\varepsilon }=\Theta \left(\left|t_{i}-t_{j}\right|-\varepsilon \right), \end{array}$$

where *i* ∈ {1,2,…, *N*_1_}, *j* ∈ {1,2,…, *N*_1_}, and Θ is a Heaviside function, which can be defined as follows:

(15) $$\begin{array}{c} \Theta \left(x\right)=\left\{\begin{array}{cc} 1, & \text{if}\;\;x\leq 0,\\ 0, & \text{else}. \end{array}\right. \end{array}$$

Thereafter, we can obtain an *N*_1_ × *N*_1_ *alignment matrix* (AM) as follows:

(16) $$\begin{array}{c} \text{AM}={\left(A_{ij}^{\varepsilon }\right)}_{N_{1}\times N_{1}}. \end{array}$$

**Step 4 .**

For the *N*_1_ × *N*_1_ elements in the alignment matrix AM, we can plot points on *i*-*j* plane for these elements in the AM with *A*_*ij*_^*ε*^ = 1 and |*i* − *j* | ≤*w*. And, for convenience, we call the obtained graph the* Alignment Scatter Diagram* (ASD) of the protein sequence pair (*s*_1_, *s*_2_).

**Figure 1:**
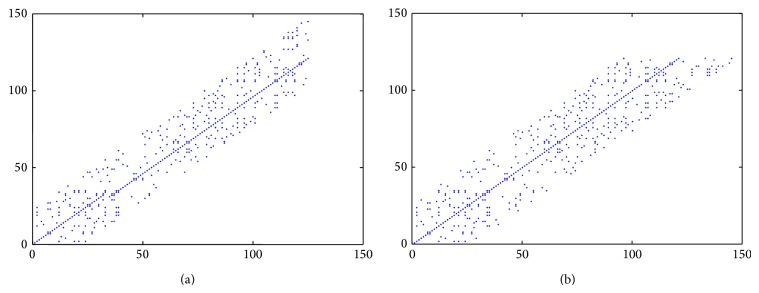
(a) The ASD of the *β*-globin protein sequence pair (chimpanzee, human) with *ξ* = 12; (b) the ASD of the *β*-globin protein sequence pair (human, gorilla) with *ξ* = 16.

For example, considering the three *β*-globin protein sequences of chimpanzee [GenBank: AAA16334.1], human [GenBank: CAA26204.1], and gorilla [GenBank: CAA43421.1] obtained from the GenBank, respectively, we illustrate the ASDs of the *β*-globin protein sequence pair (chimpanzee, human) and the *β*-globin protein sequence pair (human, gorilla) in Figures 1(a) and 1(b) separately while letting *ε* = 0.

From Figure 1, it is easy to see that there are lots of disordered points in these ASDs, which will lower the visuality of the ASDs remarkably and obstruct us from distinguishing the similarity/dissimilarity between the protein sequence pairs intuitively while observing these ASDs. Therefore, in order to improve the intuition of the ASD, we will propose a simplified variant diagram of the ASD, which is called the* Alignment Diagonal Line Diagram* (ADLD).

For convenience, in an ASD, we call its main diagonal line the* artery tracks* (ATs) and the lines parallelling to its main diagonal line the* by-path tracks* (BTs), respectively. And, in addition, we define a set consisting with no less than *δ* consecutive APs on the AT or BTs as a CAPS, where *δ* ≥ 1 is a given threshold.

For a given CAPS caps_1_, if there is no CAPS caps_2_ satisfying caps_1_ ⊂ caps_2_, then we call the caps_1_ a maximum CAPS. And, for convenience, we call the line formed by connecting all of the APs in a maximum CAPS a* similar fragment* (SF), and simultaneously we call all of the APs on the AT but not on any SFs the* free points* (FPs).

Obviously, in an ASD, if keeping all of the SFs and FPs only and omitting all those other APs, then we will obtain a simplified variant diagram of the ASD, and, for convenience, we call it the* Alignment Diagonal Line Diagram* (ADLD). Apparently, if *δ* = 1, then an ADLD will degenerate into an ASD. Therefore, in actual applications, we suggest that *δ* will be no less than 2. And, particularly, in order to find more accurate SFs in the ADLD of a protein sequence pair, the longer the protein sequences in the protein sequence pair are the bigger the value of *δ* shall be.

For convenience of analysis, in an ADLD, suppose that there are *K*_1_ different SFs and *K*_2_ different FPs on its AT, *K* different BTs locating above its AT, and *K* different BTs locating below its AT; then we get the following.

1. For these *K*_1_ different SFs and *K*_2_ different FPs on the AT of the ADLD, we will number these *K*_1_ SFs and *K*_2_ FPs from left to right and utilize {ASF^1^, ASF^2^,…, ASF^*K*_1_^} and {FP^1^, FP^2^,…, FP^*K*_2_^} to represent these *K*_1_ SFs and *K*_2_ FPs separately. And, in addition, we would also call these SFs on the AT of the ADLD the ASFs.
2. For these *K* different BTs locating above the AT, we will number these BTs from down to up and utilize {BT_1_, BT_2_,…, BT_*K*_} to represent these BTs separately, and, for these *K* different BTs locating below the AT, we will number these BTs from up to down and utilize {BT_−1_, BT_−2_,…, BT_−*K*_} to represent these BTs separately.
3. For each BT_*l*_, where *l* ∈ {1,2,…, *K*}, suppose that there are *K*_3_ different SFs on the BT_*l*_; then we will number these *K*_3_ SFs from left to right and utilize {BSF_*l*_^1^, BSF_*l*_^2^,…, BSF_*l*_^*K*_3_^} to represent these SFs separately. And, in addition, we would also call these SFs on the BTs of the ADLD the BSFs.

**Figure 2:**
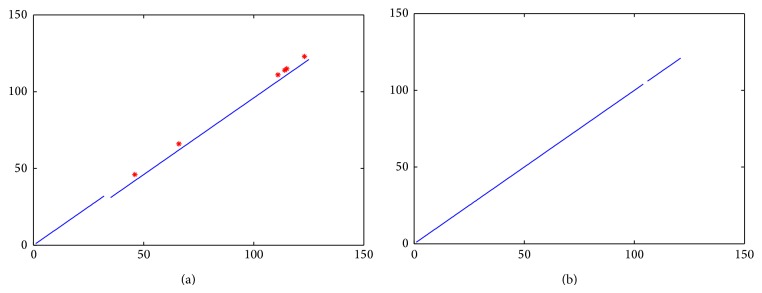
(a) The ADLD of the protein sequence pair (chimpanzee, human); (b) the ADLD of the protein sequence pair (human, gorilla).

According to the above assumptions, in Figure 2, we show the two ADLDs corresponding to the ASDs illustrated in Figures 1(a) and 1(b) while letting *δ* = 3. And, in addition, to make the ADLDs more visual and intuitional, in Figure 2, we use the red “∗” to represent the FPs on the AT and the blue lines to represent the SFs on the AT or BTs.

From Figure 2(a), it is easy to see that there are two SFs in the ADLD of the sequence pair (chimpanzee, human); one is ASF^1^, that is, the line segment from the point (1,1) to the point (32,32), and the other is BSF_−4_^1^, that is, the line segment from the point (35,31) to the point (125,121). And, in addition, there are totally 6 FPs in the ADLD, which are FP^1^(46,46), FP^2^(66,66), FP^3^(111,111), FP^4^(114,114), FP^5^(115,115), and FP^6^(123,123), respectively.

Observing Figure 2(b), we can easily find that there are also two SFs in the ADLD of the sequence pair (human, gorilla). But, different from that in Figure 2(a), the two SFs in Figure 2(b) are both ASFs; one is ASF^1^, that is, the line segment from the point (1,1) to the point (104,104), and the other is ASF^2^, that is, the line segment from the point (106,106) to the point (121,121). And, in addition, the two ASFs in Figure 2(b) are separated by one gap, and there exist no FPs or BSFs on the AT or BTs.

Through analysis, we can know that, for a given protein sequence pair, if there exist some deletions or insertions of amino acid segments between the two protein sequences, then there will exist some misalignments of SFs in their ADLD; that is, some ASFs on the AT will be transformed into BSFs on some BTs. And, in addition, if there exist some substitutions of the amino acids between the two protein sequences, then, in their ADLD, there will exist some gaps between two neighboring SFs or FPs on the AT. Furthermore, if there exist some insertions, deletions, or substitutions of the amino acid segments at the end of the two protein sequences, then, in their ADLD, there will exist no SFs or FPs on the AT or BTs.

From the above descriptions, it is easy to know that the ADLD of any given protein sequence pair obtained by our above proposed method reflects some inner and specific differences between these two protein sequences in the given protein sequence pair, which may be useful in the similarity/dissimilarity analysis of protein sequence pairs.

## 3. Results and Discussion

### 3.1. Method for Similarity/Dissimilarity Analysis of Protein Sequences Based on the ADLDs

According to the above analysis, we have known that the ADLDs may be useful in analyzing the differences of the inner structures of protein sequence pairs. In this section, we will show how to utilize the ADLDs to analyze the similarity/dissimilarity of a group of protein sequences.

Generally, suppose that there are *N* protein sequences {Ψ_1_, Ψ_2_,…, Ψ_*N*_}; then while applying the ADLDs to analyze the similarity/dissimilarity of these *N* sequences, the similarity/dissimilarity matrix of these *N* sequences can be obtained through the following steps.

**Step 1 .**

According to the method given in Section 2.5, transform these *N* protein sequences into *N* numerical sequences {*S*_1_, *S*_2_,…, *S*_*N*_}.

**Step 2 .**

For a given protein sequence pair {Ψ_*a*_, Ψ_*b*_}, *a* ∈ {1,2,…, *N*}, *b* ∈ {1,2,…, *N*}, we can obtain their ADLD through adopting the method proposed in Section 2.6, and then we can obtain all of the SFs (including ASFs and BSFs) and FPs in the ADLD. Hence, we can obtain the lengths of these ASFs, the lengths of these BSFs, and the number of these FPs, respectively.

**Step 3 .**

Suppose that there are totally *L*_1_ different ASFs such as {ASF^1^, ASF^2^,…, ASF^*L*_1_^}, *L*_2_ different BSFs such as {BSF_*l*_1__^1^, BSF_*l*_2__^2^,…, BSF_*l*_*L*_2___^*L*_2_^}, and *L*_3_ different FPs such as {FP^1^, FP^2^,…, FP^*L*_3_^} in the ADLD. And, in addition, for each ASF^*i*^ and BSF_*l*_*j*__^*j*^, let their length be length^*i*^ and length_*j*_, respectively, where *i* ∈ {1,2,…, *L*_1_} and *j* ∈ {1,2,…, *L*_2_}; then we can define the* similarity degree* (SD) of {Ψ_*a*_, Ψ_*b*_} as follows:

(17) $$\begin{array}{c} \text{SD}\left(\Psi_{a},\Psi_{b}\right)=\overset{L_{1}}{\underset{i=1}{\sum }}{\text{length}}^{i}+\overset{L_{2}}{\underset{j=1}{\sum }}{\text{length}}_{j}+L_{3}. \end{array}$$

And, therefore, according to these *N* protein sequences {Ψ_1_, Ψ_2_,…, Ψ_*N*_}, we can obtain an *N* × *N matching matrix* (MM) as follows:

(18) $$\begin{array}{c} \text{MM}={\left[d_{ij}\right]}_{N\times N}, \end{array}$$

where

(19) dij=SDΨi,Ψj,if  i≥j,0,else,for  i∈1,2,…,N, j∈1,2,…,N.

**Step 4 .**

Based on the matching matrix MM and all of its components *d*_*ij*_, where *i* ∈ {1,2,…, *N*} and *j* ∈ {1,2,…, *N*}, then we can obtain an *N* × *N similarity/dissimilarity matrix* (SM) of these *N* protein sequences {Ψ_1_, Ψ_2_,…, Ψ_*N*_} as follows:

(20) $$\begin{array}{c} \text{SM}={\left[s_{ij}\right]}_{N\times N}, \end{array}$$

where

(21) sij=1−Λdijdii,if  i≥j,0,else,Λdijdii=1,if  dijdii≥1,dijdii,else,for  i∈1,2,…,N, j∈1,2,…,N.

According to the above steps, we present an example through implementing the ADLDs to analyze the similarity/dissimilarity of 16 ND5 proteins (illustrated in Table 5) while letting *δ* = 3 and illustrate the results of similarity/dissimilarity matrix in Table 6.

Observing Table 6, it is easy to find that there are some similar pairs such as (c-chim, pi-chim) with the distance** 0.0510**, (human, c-chim) with the distance** 0.0814**, (human, pi-chim) with the distance** 0.0720**, (gorilla, c-chim) with the distance** 0.0865**, (gorilla, pi-chim) with the distance** 0.0833**, and (fin-whale, blue-whale) with the distance** 0.0324**. And, among them, the opossum seems to be a peculiar mammal, since the shortest distance between it and the remaining mammals is more than** 0.4023**. Obviously, the result is consistent with the fact that opossum is the most remote species from the remaining mammals.

Additionally, gallus seems to be more peculiar than opossum, since the shortest distance between it and the remaining animals is more than** 0.4423**, which is bigger than** 0.4023** (the shortest distance between Opossum and the remaining mammals). Obviously, the result is consistent with the fact that gallus is not a kind of mammal.

Therefore, it is apparent that the results illustrated in Table 6 are wholly consistent with the results of the known fact of evolution. That is to say, our ADLDs based method can be utilized as an effective way to analyze the similarities/dissimilarities of protein sequences.

### 3.2. The Phylogenetic Tree of the Protein Sequences Based on the ADLDs

A* phylogenetic tree* is a diagram that is used to represent the evolutionary relationships of organisms that are thought to have a common ancestry, and it is a commonly used tool for researchers in some fields to help them analyze the clustering of different species.

**Figure 3:**
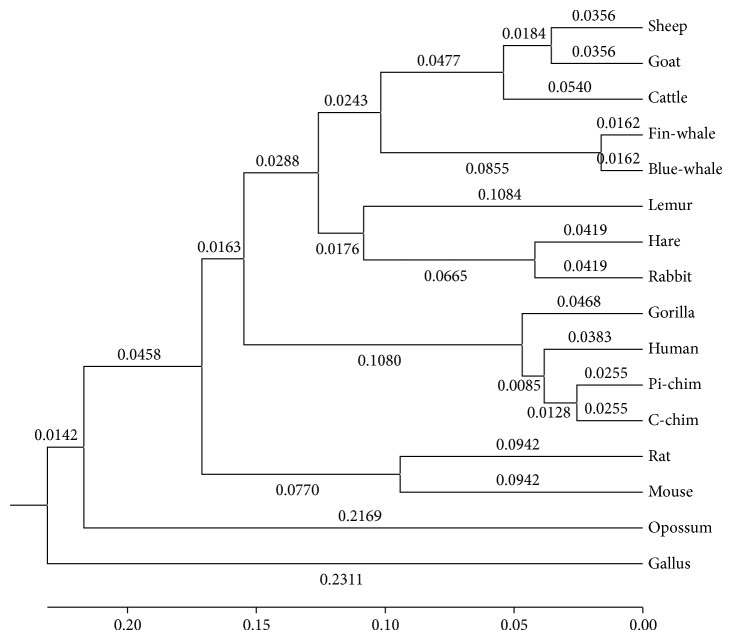
The phylogenetic tree of the 16 species based on the ADLDs based method.

Obviously, only through observing the similarity/dissimilarity matrix illustrated in Table 6, we will find that it is not very convenient to distinguish the similarity/dissimilarity of protein sequences. Therefore, in order to show the similarity/dissimilarity of the protein sequences more vividly and intuitively, according to the similarity/dissimilarity matrix illustrated in Table 6, then we will construct the phylogenetic tree of the above 16 ND5 proteins through adopting the software MEGA 6.06 that is provided by Tamura et al. [41], and the result is illustrated in Figure 3.

From Figure 3, it is obvious that we can not only find out the evolutionary relationships of these 16 ND5 protein sequences visually and intuitively but also know easily that the constructed phylogenetic tree is consistent with the results of the known fact of evolution to some degree.

To further validate the performance of our ADLDs based method, we applied our method to analyze the similarity/dissimilarity of another group of proteins including 29 spike proteins of coronavirus and compared our method with the method proposed by Wen and Zhang [17] based on the above given 16 ND5 proteins and the following 29 spike proteins, respectively. The basic information of the 29 spike proteins is illustrated in Table 7.

**Figure 4:**
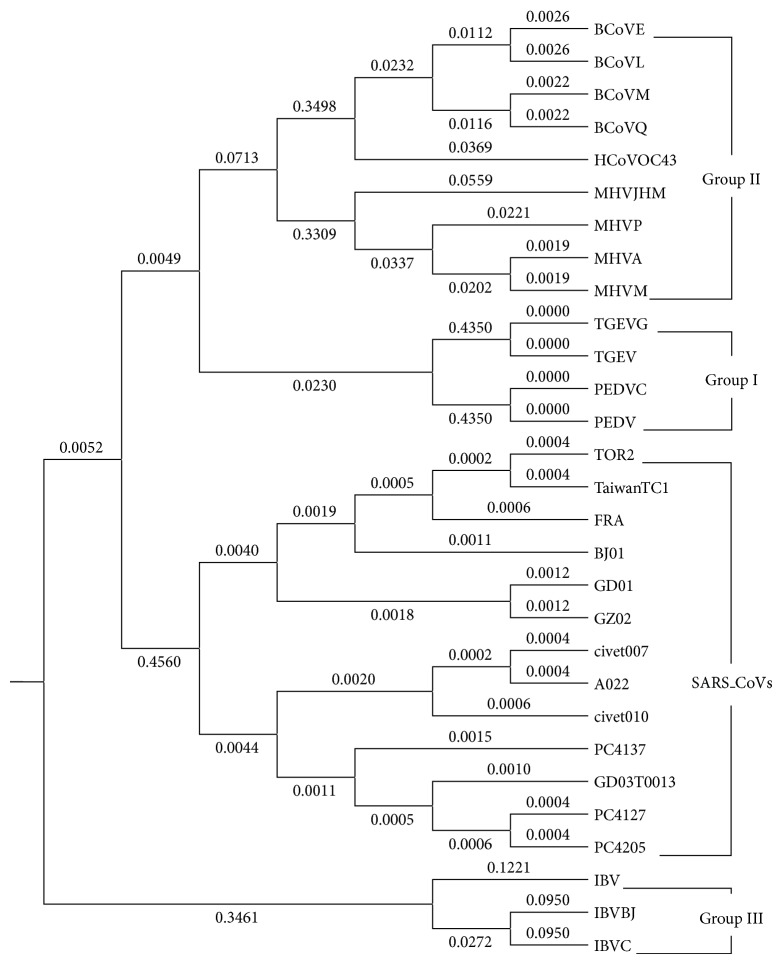
The phylogenetic tree of the 29 spike proteins of coronavirus constructed by adopting the ADLDs based method with *δ* = 5.

For the 29 spike proteins illustrated in Table 7, we construct the phylogenetic tree in Figure 4. Since the spike protein sequences are very long (with more than 1100 amino acids), therefore, during simulation, we set *δ* = 5 to avoid the effect of noise points.

Generally,* coronavirus* can always be classified into four classes such as the Group I, the Group II, the Group III, and the SARS-CoVs (Severe Acute Respiratory Syndrome Coronaviruses). And, among these four classes, the Group I includes the* Canine coronavirus* (CCoV), the* Feline coronavirus* (FCoV), the* Human coronavirus* 229E (HCoV-229E), the* Porcine epidemic diarrhea virus* (PEDV), and the* Transmissible gastroenteritis virus* (TGEV). The Group II includes the* Bovine coronavirus* (BCoV),* Human coronavirus* OC43 (HCoV-OC43), the* Murine coronavirus,* Mouse hepatitis virus (MHV), the Porcine hemagglutinating encephalomyelitis virus (HEV), and the* Rat coronavirus* (RtCoV). The Group III contains the* Avian infectious bronchitis virus* (IBV) and the* Turkey coronavirus* (TCoV).

From observing Figure 4, it is easy to know that the 29 spike proteins of coronavirus can be perfectly classified into the above four classes by our ADLDs based method.

**Figure 5:**
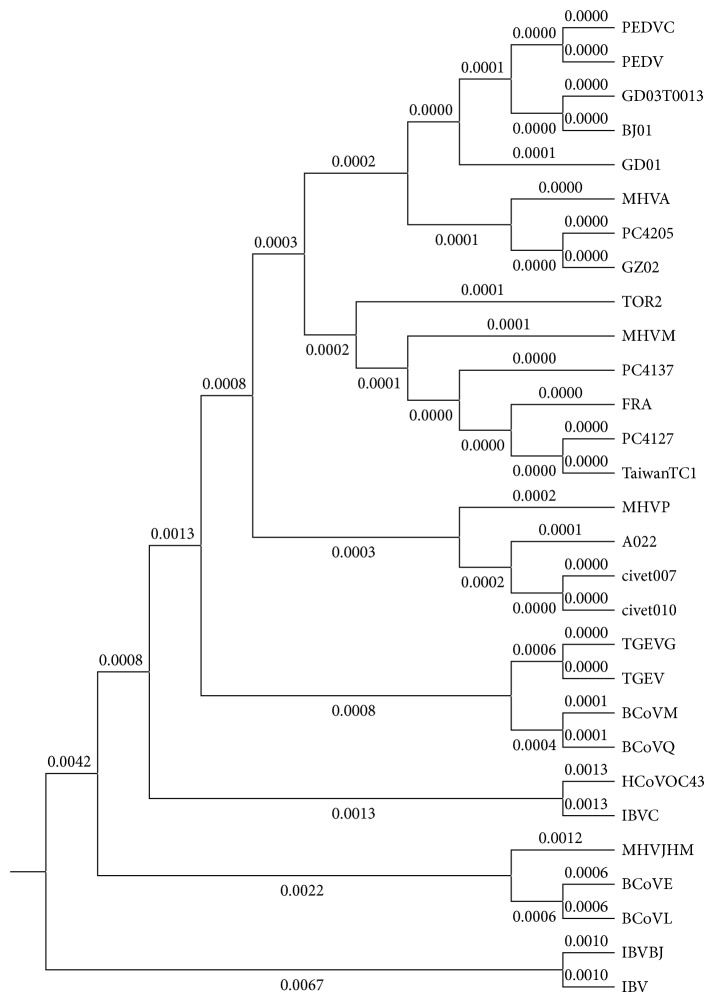
The phylogenetic tree of the 29 spike proteins of coronavirus constructed by adopting the method proposed by Wen and Zhang.

**Figure 6:**
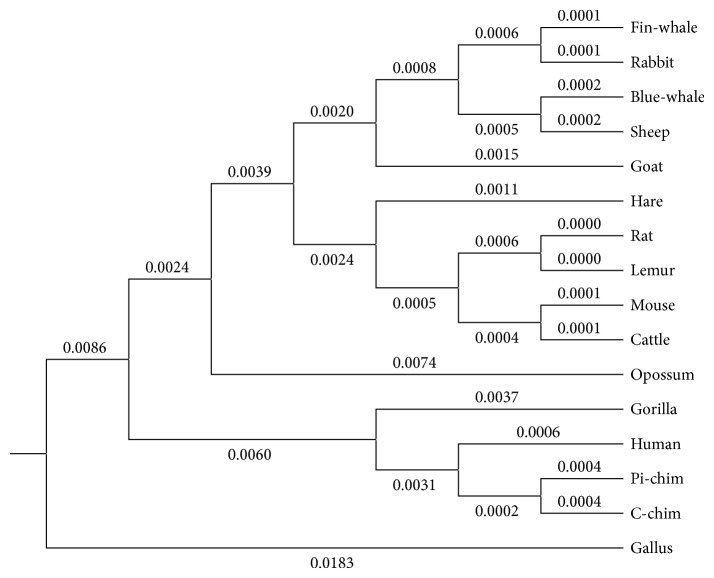
The phylogenetic tree of the 16 ND5 proteins constructed by adopting the method proposed by Wen and Zhang.

Finally, for the convenience of comparison, we illustrate the phylogenetic trees of the above given 29 spike proteins of* coronavirus* and 16 ND5 proteins, constructed by adopting the method proposed by Wen and Zhang [17], in Figures 5 and 6, respectively.

Comparing Figure 3 with Figure 6 and Figure 4 with Figure 5, respectively, it is obvious that the phylogenetic trees obtained by the method proposed by Wen and Zhang are quite unreasonable and not consistent with the known facts of evolution at all. But, on the contrary, the phylogenetic trees obtained by our ADLDs based method are not only quite reasonable but also consistent with the known facts of evolution to some degree. Therefore, there is no doubt that the performance of our method is much better than that of the method proposed by Wen and Zhang.

### 3.3. The Analysis of Intuition and Visuality of the ADLDs

In Section 2.6, we have stated that the ADLDs of protein sequence pairs are intuitional and visual. In this section, we will further discuss the intuition and visuality of the ADLDs in detail.

**Figure 7:**
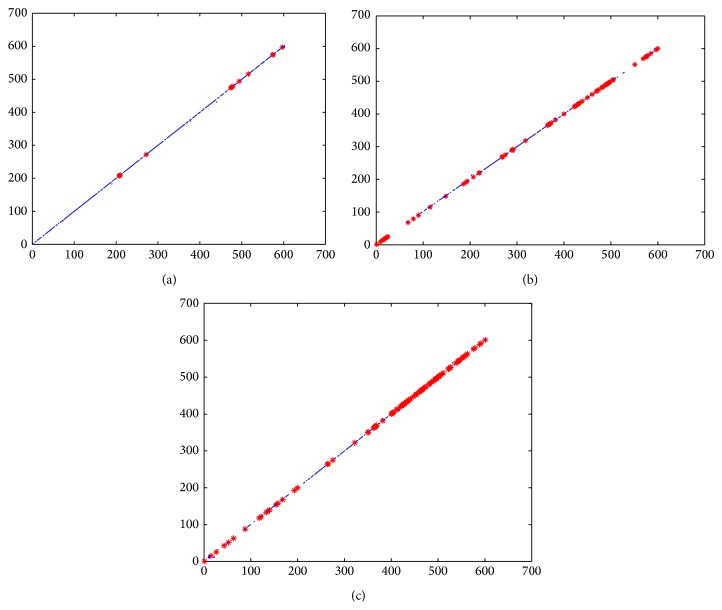
(a) The ADLD of the similar pair (human, gorilla); (b) the ADLD of the dissimilar pair (human, opossum); (c) the ADLD of the dissimilar pair (human, gallus).

From Table 6, we can obtain some similar pairs such as (fin-whale, blue-whale), (pi-chim, c-chim), (Human, c-chim), (cheep, goat), (human, pi-chim), and (hare, rabbit) and some dissimilar pairs such as (human, opossum) and (human, gallus), among the above given 16 ND5 proteins. From these similar/dissimilar pairs, we will choose three pairs including (human, gorilla), (human, opossum), and (human, gallus) as examples to further show the intuition and visuality of the ADLDs of these three protein sequence pairs. The ADLDs of these three similar/dissimilar pairs are illustrated in Figure 7, while letting *δ* = 3.

Observing Figure 7, we can clearly find that the total length of all of the SFs in each of these three ADLDs satisfies* the total length of all of the SFs in the ADLD of Figure 7(a) > the total length of all of the SFs in the ADLD of Figure 7(b) > the total length of all of the SFs in the ADLD of Figure 7(c)*. Therefore, we can intuitively identify that the similarity of the proteins in each of these three protein sequence pairs satisfies* the similarity of the proteins in the pair (human, gorilla) > the similarity of the proteins in the pair (human, opossum) > the similarity of the proteins in the pair (human, gallus)*.

Moreover, from Figure 7, we can also intuitively identify that the two protein sequences in the protein sequence pair (human, gorilla) are very similar to each other, since the total length of all of the SFs in the ADLD of Figure 7(a) looks very long. But, on the contrary, we can intuitively identify that the two protein sequences in either the protein sequence pair (human, opossum) or the protein sequence pair (human, gallus) are apparently dissimilar to each other, since both the total length of all of the SFs in the ADLD of Figure 7(b) and that in the ADLD of Figure 7(c) look very short.

And, through statistic, we can know that the actual total lengths of all of the SFs in the ADLDs of these three protein sequence pairs (human, gorilla), (human, opossum), and (human, gallus) are 556, 288, and 248, respectively.

Additionally, observing Figures 2(a) and 2(b), hardly can we distinguish the total length of all of the SFs (including ASFs and BSFs) in the ADLD of Figure 2(a) and that in the ADLD of Figure 2(b), since the total lengths of all of the SFs in these two ADLDs look nearly the same. And, through statistic, we can know that the actual total lengths of all of the SFs in the ADLDs of Figures 2(a) and 2(b) are 123 and 120, respectively, and are really close to each other. But, through comparing Figure 2(a) with Figure 2(b) more carefully, we can further discover that, different from Figure 2(b), except for the SFs, there are also 6 different FPs in the ADLD of Figure 2(a), while there are no FPs in the ADLD of Figure 2(b); therefore, we can intuitively identify that the two protein sequences in the protein sequence pair (chimpanzee, human) are more similar to the two protein sequences in the protein sequence pair (human, gorilla).

Hence, from the above descriptions, we can know that the ADLDs obtained by our newly proposed method are quite visual and intuitional and may be a powerful and effective tool for visual comparison of protein sequences and numerical sequences in other research fields.

## 4. Conclusions

In this paper, a novel ADLDs based graphical representation of protein sequences is proposed, which is utilized to analyze the similarity/dissimilarity of protein sequences. To validate the performances of the new method, we select two groups of well-known protein sequences as examples, and, additionally, in order to observe the similarity/dissimilarity of protein sequences more intuitively, we construct the phylogenetic trees of protein sequences. The results show that our ADLDs based method not only has good performances and effects in the similarity/dissimilarity analysis of protein sequences but also does not require complex computation, since there are no high dimensional matrixes required. Therefore, it means that our ADLDs based method can work well in the analysis of protein sequences.

## Figures and Tables

**Table 1:** The full list of 20 amino acids and the value of their 9 different properties.

Amino acid	Symbol	mW	hI	pK1	pK2	pI	*S*	cN	*F* (%)	vR
Alanine	A	89.079	1.8	2.34	9.69	6.01	167.2	4	7.8	67
Cysteine	C	121.145	2.5	1.96	10.28	5.07	0	2	1.9	86
Aspartic acid	D	133.089	−3.5	1.88	9.6	2.77	5	2	5.3	91
Glutamic acid	E	147.116	−3.5	2.19	9.67	3.22	8.5	2	6.3	109
Phenylalanine	F	165.177	2.8	1.83	9.13	5.48	27.6	2	3.9	135
Glycine	G	75.052	−0.4	2.34	9.6	5.97	249.9	4	7.2	48
Histidine	H	155.141	−3.2	1.82	9.17	7.59	0	2	2.3	118
Isoleucine	I	131.16	4.5	2.36	9.68	6.02	34.5	3	5.3	124
Lysine	K	146.17	−3.9	2.18	8.95	9.74	739	2	5.9	135
Leucine	L	131.16	3.8	2.36	9.6	5.98	21.7	6	9.1	124
Methionine	M	149.199	1.9	2.28	9.21	5.74	56.2	1	2.3	124
Asparagine	N	132.104	−3.5	2.02	8.8	5.41	28.5	2	4.3	96
Proline	P	115.117	1.6	1.99	10.96	6.48	1620	4	5.2	90
Glutamine	Q	146.131	−3.5	2.17	9.13	5.65	7.2	2	4.2	114
Arginine	R	174.188	−4.5	2.17	9.04	10.76	855.6	6	5.1	148
Serine	S	105.078	−0.8	2.21	9.15	5.68	422	6	6.8	73
Tyrosine	T	119.105	−0.7	2.11	9.62	5.87	13.2	4	5.9	93
Valine	V	117.133	4.2	2.32	9.62	5.97	58.1	4	6.6	105
Tryptophan	W	204.213	−0.9	2.38	9.39	5.89	13.6	1	1.4	163
Threonine	Y	181.176	−1.3	2.2	9.11	5.66	0.4	2	3.2	141

**Table 2:** The 9 eigenvalues (λ) of **R** and the contribution rates (CR) and the accumulative contribution rates (ACR) of the 9 principal components obtained by conducting PCA of the 20 amino acids.

Number	*λ*	CR	ACR
1	3.2237	0.3582	0.3582
2	1.9132	0.2126	0.5708
3	1.4048	0.1561	0.7269
4	1.1876	0.1320	**0.8588**
5	0.4959	0.0551	0.9139
6	0.4467	0.0496	0.9635
7	0.1992	0.0221	0.9857
8	0.1218	0.0135	0.9992
9	0.0071	0.0008	1.0000

**Table 3:** The 4 eigenvectors {*a*_1_, *a*_2_, *a*_3_, *a*_4_} corresponding to the first 4 eigenvalues in Table 2.

*a*_1_	*a*_2_	*a*_3_	*a*_4_
**0.5036**	0.1436	0.0571	0.2158
−0.2454	−0.1875	0.2304	**0.6547**
−0.1634	0.1820	**0.6298**	0.2288
−0.3101	−0.1883	−0.3964	**0.5071**
0.0702	**0.6464**	−0.0786	0.0532
−0.1665	**0.4465**	**−0.5280**	0.1877
−0.3872	**0.3931**	0.1003	−0.0532
**−0.4377**	0.1844	0.2544	−0.2273
**0.4349**	0.2643	0.1738	0.3495

**Table 4:** The total scores of the 20 amino acids.

Symbols of amino acids	Total scores
A	−0.9324
C	−0.5985
D	−0.6709
E	−0.2296
F	0.4298
G	−1.1780
H	0.4476
I	0.1435
K	0.7868
L	−0.1205
M	0.5735
N	−0.0242
P	−0.9822
Q	0.2848
R	1.1169
S	−0.7077
T	−0.4525
V	−0.2643
W	1.4729
Y	0.9050

**Table 5:** The basic information of 16 ND5 protein sequences.

Number	Name	Abbreviation	Access number	Length
1	Human	Human	ADT80430.1	603
2	Gorilla	Gorilla	NP_008222	603
3	Pigmy chimpanzee	Pi-chim	NP_008209	603
4	Common chimpanzee	C-chim	NP_008196	603
5	Fin-whale	Fin-whale	NP_006899	606
6	Blue-whale	Blue-whale	NP_007066	606
7	Rat	Rat	AP_004902.1	610
8	Mouse	Mouse	NP_904338	607
9	Opossum	Opossum	NP_007105	602
10	Sheep	Sheep	ABW22903.1	606
11	Goat	Goat	BAN59258.1	606
12	Lemur	Lemur	CAD13431.1	603
13	Cattle	Cattle	ADN11902.1	606
14	Hare	Hare	CAD13291.1	603
15	Gallus	Gallus	BAE16036.1	605
16	Rabbit	Rabbit	NP_007559.1	603

**Table 6:** The similarity/dissimilarity matrix for the 16 ND5 proteins based on the ADLDs based method.

	Human	Gorilla	Pi-chim	C-chim	Fin-whale	Blue-whale	rat	mouse	opossum	sheep	goat	lemur	cattle	hare	gallus	rabbit
Human	0.0000															
Gorilla	0.1111	0.0000														
Pi-chim	**0.0720**	**0.0833**	0.0000													
C-chim	**0.0814**	**0.0865**	**0.0510**	0.0000												
Fin-whale	0.3396	0.3285	0.3222	0.3301	0.0000											
Blue-whale	0.3474	0.3333	0.3285	0.3301	**0.0324**	0.0000										
Rat	0.3693	0.3622	0.3636	0.3716	0.3333	0.3381	0.0000									
Mouse	0.3740	0.3686	0.3716	0.3748	0.3317	0.3333	0.1883	0.0000								
Opossum	0.4476	0.4551	0.4290	0.4418	0.4515	0.4519	0.4513	0.4479	0.0000							
Sheep	0.3020	0.2933	0.2871	0.2951	0.2023	0.2067	0.3149	0.3219	0.4121	0.0000						
Goat	0.3036	0.2901	0.2871	0.2919	0.1958	0.2147	0.3166	0.3468	0.4202	**0.0712**	0.0000					
Lemur	0.2989	0.2708	0.2839	0.2967	0.2557	0.2724	0.3166	0.3670	0.4055	0.2087	0.2317	0.0000				
Cattle	0.3114	0.3045	0.3046	0.3062	0.1958	0.2051	0.3149	0.3173	0.4235	**0.0906**	0.1254	0.2184	0.0000			
Hare	0.3146	0.3157	0.3062	0.3046	0.2832	0.2788	0.3166	0.3421	0.4023	0.2217	0.2508	0.2053	0.2532	0.0000		
Gallus	0.4726	0.4920	0.4737	0.5008	0.4450	0.4423	0.4903	0.4743	0.4691	0.4239	0.4524	0.4680	0.4183	0.4660	0.0000	
Rabbit	0.3255	0.3189	0.3222	0.3142	0.2896	0.2756	0.3084	0.3390	0.4332	0.2184	0.2603	0.2282	0.2612	**0.0837**	0.4434	0.0000

**Table 7:** The basic information of 29 spike proteins.

Number	Access number	Abbreviation	Length
1	CAB91145	TGEVG	1447
2	NP_058424	TGEV	1447
3	AAK38656	PEDVC	1383
4	NP_598310	PEDV	1383
5	NP_937950	HCoVOC43	1361
6	AAK83356	BCoVE	1363
7	AAL57308	BCoVL	1363
8	AAA66399	BCoVM	1363
9	AAL40400	BCoVQ	1363
10	AAB86819	MHVA	1324
11	YP_209233	MHVJHM	1376
12	AAF69334	MHVP	1321
13	AAF69344	MHVM	1324
14	AAP92675	IBVBJ	1169
15	AAS00080	IBVC	1169
16	NP_040831	IBV	1162
17	AAS10463	GD03T0013	1255
18	AAU93318	PC4127	1255
19	AAV49720	PC4137	1255
20	AAU93319	PC4205	1255
21	AAU04646	civet007	1255
22	AAU04649	civet010	1255
23	AAV91631	A022	1255
24	AAP51227	GD01	1255
25	AAS00003	GZ02	1255
26	AAP30030	BJ01	1255
27	AAP50485	FRA	1255
28	AAP41037	TOR2	1255
29	AAQ01597	TaiwanTC1	1255
